# Cost-effectiveness analysis of the available pneumococcal conjugated vaccines for children under five years in Colombia

**DOI:** 10.1186/s12962-015-0032-1

**Published:** 2015-04-10

**Authors:** Jaime E Ordóñez, John Jairo Orozco

**Affiliations:** HEMO Group Carrera 25A N° 1A Sur-45, piso 5.Torre Médica El Tesoro Medellín, Medellín, Colombia; CES University, Medellín, Colombia

**Keywords:** 13-valent pneumococcal vaccine, 10-valent pneumococcal vaccine, Pneumococcal infections, Infant, Cost-benefit analysis, Child mortality

## Abstract

**Background:**

Pneumococcal diseases in children under five years are common and preventable. In Colombia there are two pneumococcal conjugate vaccines (PCV) that have proved clinical efficacy. The aim was to estimate the cost-effectiveness of 13-valent PCV (PCV13) and 10-valent PCV (PCV10) in terms of prevention of Invasive Pneumococcal Diseases (IPD), radiologically-confirmed pneumonia, and their related mortality, as well as, acute otitis media (AOM) in a cohort of newborns in Colombia.

**Methods:**

We developed an analytical decision tree model with national data including the distribution of pneumococcal serotypes in Colombia between 2009 and 2013. A simulation of vaccination of 90% of newborns in Colombia took place with a time horizon of 5 years. The analysis was done from the Colombian health system perspective. Vaccines efficacy parameters were measured as life-years gained (LYG) and avoided morbidity by pneumococcal diseases; they were determined by a systematic review of literature. A health insurance company provided the costs. A probabilistic and a univariate sensitivity analysis for epidemiological, efficacy and cost parameters were done.

**Results:**

After 5 years projection, PCV13 would prevent 437 deaths due to pneumococcal infections versus 321 that would be prevented by PCV10, compared to no vaccination. PCV13 would generate 25 396 LYG, and PCV10 would generate 18 708 LYG. Medical costs avoided would be US$ 19 479 395 for PCV13 and US$ 13 703 271 for PCV10. Compared to no vaccination, PCV13 and PCV10 were cost-effective, with an incremental cost-effectiveness ratio (ICER) of US$ 489.26 and US$ 813.41 per additional LYG, respectively; besides, PCV13 was dominant over PCV10 due to lower costs and better outcomes.

**Conclusion:**

PCV13 is a cost-saving strategy compared with PCV10, as part of a universal coverage vaccination program in Colombian children under one year. PCV13 is expected to lead to a greater decrement in infant mortality from pneumococcal diseases, and a higher cost saving by preventing more pneumococcal diseases compared with PCV10 in a 5 years projection.

## Introduction

The diseases produced by *Streptococcus Pneumoniae* (SP), such as pneumonia, sepsis (including bacteremia), meningitis and acute otitis media (AOM) are a severe public health problem. Annually, 2 million children die worldwide due to pneumonia, which is more than those due to AIDS, malaria and measles together [1]. In order to reduce the mortality rate in children younger than 5 years by two thirds, one of the Millennium Development Goals (MDG) [2] included strategies as pneumococcal vaccination. [3].

In 2000 was introduced the first pneumococcal conjugated vaccine. This vaccine was composed of purified capsular polysaccharides of seven pneumococcus serotypes (4, 6B, 9 V, 14, 18C, 19 F and 23 F) conjugated to a diphteria protein (CRM_197_), and demonstrated its clinical efficacy and effectiveness (PCV-7) [4-6]. A health economics analysis concluded that PCV7 would prevent 678,000 AOM cases and 175,000 pneumonia cases due to SP by year in Latin America and the Caribbean region [7]. In Uruguay, PCV7 was found highly cost-effective and recommendable for countries with a similar distribution of serotypes [8].

In 2010 it was launched, a 10-valent conjugate vaccine (PCV-10), that add the three serotypes 1, 5 and 7 F. Eight of the ten serotypes in PCV10 are conjugated to the D protein of non typeable *Haemophilus influenza*, of the remaining two, one to tetanus toxoid and one to diphtheria toxoid. Later, the 13-valent pneumococcal conjugate vaccine (PCV13) was launched with the three additional serotypes 3, 6A and 19A. All 13 serotypes in PCV13 are conjugated to CRM_197_, the same protein carrier used for PCV7.

Currently, in Colombia there are two available technologies to prevent pneumococcal diseases in children younger than 5 years, PCV10 and PCV13. Due to the difference in terms of the effectiveness and costs of the vaccines, it is necessary to estimate the cost-effectiveness of both strategies in order to allow an informed decision taking. Our objective is to estimate the cost-effectiveness ratio between PCV13 and PCV10 as part of an infant vaccination program, considering the current pneumococcal serotypes distribution in Colombia.

## Methods

**Figure 1:**
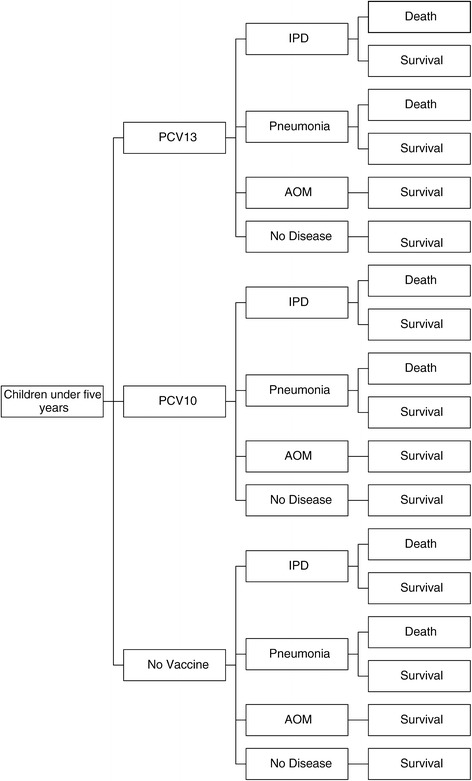
**Decision tree for the cost-effectiveness analysis on vaccinating with PCV13, PCV10 or not vaccinating in a cohort of newborns in Colombia.**

An analytical decision tree model was constructed (Figure 1) with local data on the prevalence of Invasive Pneumococcal Disease (IPD) (meningitis and sepsis, including bacteremia), pneumococcal AOM and radiologically-confirmed pneumonia. The model was developed by the authors and simulates a cohort of newborns in Colombia. The model assumed that at the beginning, individuals would be vaccinated with PCV13 or PCV10 or would not be vaccinated. Mortality rates from IPD and pneumonia were considered to calculate deaths avoided. Costs of medical treatments were calculated based on the site of healthcare, it means, inpatient or outpatient.

To determine epidemiologic distribution of SP serotypes found in children younger than 5 years in Colombia between 2009 and 2013, we used the data of Regional Vaccine System (SIREVA II; by their acronym in Spanish), which reports serotypes of all isolates made in Colombia by IPD. A systematic review was done, in order to determine the efficacy of both vaccines, adjusted to the distribution of pneumococcal serotypes. This research does not involve any human subject, and secondary sources of information such as scientific papers and bills payment were used.

### Model

The cohort to evaluate morbidity children was born in Colombia in 2012; with a 5 follow up years. In order to calculate the life-years gained (LYG), the life expectancy during the period 2010-2015 for both sexes was assumed [9]. The reason for using children born in 2012 as the cohort group rather than the population projections made by the National Administrative Department of Statistics (DANE; by their acronym in Spanish) for later years; is that the results of those projections are 20% below of the real-life data.

The international health agencies and the local regulatory agency (INVIMA for Colombia) have approved the use of both vaccines: PCV10 and PCV13, based on comparison of immunologic response to PCV7 to common serotypes. Thus, PCV7 is the basis for the clinical efficacy and is the support to approve other conjugated vaccines [10].

Since PCV13 and PCV10 have regulatory approval to prevent pneumococcal diseases caused by the serotypes included in the vaccines, the probabilities of becoming ill by these serotypes were adjusted according to the SIREVA II, which reports the serotypes that cause IPD and are isolated in Colombia [11].

In order to depict the risk for clinical outcomes, pneumococcal sepsis, pneumococcal meningitis, radiologically-confirmed pneumonia and pneumococcal AOM, a decision analytical tree model was used [4-8]. To estimate avoided deaths, the probability of death from pneumonia, meningitis and sepsis was considered. A discount rate of 3% for costs and effects was applied, according to the recommendation of the World Health Organization (WHO) [12]. The analysis was done from the Colombian health system perspective.

Three alternatives were considered: not vaccination, vaccination with PCV10 or vaccination with PCV13. The direct efficacy of the vaccines was considered during the first 5 years of life. The cost-effectiveness analysis was performed among the three alternatives in terms of each disease related morbidity cases and deaths, as well as, life-years gained (LYG).

### Epidemiologic parameters

In order to determine the likelihood of IPD, the incidence was calculated using a local study performed in Colombian population [13]. The likelihood of developing radiologically-confirmed pneumonia was calculated [14] based on population indicators of pneumonia like the discharge diagnosis in children younger than 5 years in Medellin during 2009 [15]. This was adjusted according to the proportion of children with a clinical diagnosis of pneumonia that could be confirmed radiologically [13].

The incidence of AOM was estimated only for pneumococcal AOM according with the indications approved by the INVIMA. The population indicators of AOM used were the discharge diagnosis from the emergency room or outpatient visits in children younger than 5 years in Medellin during 2009 [15]. This was adjusted according to the proportion of children with a clinical diagnosis of AOM who had pneumococcus as the confirmed etiologic agent [17].

The probability of mortality due to IPD was 37% and to pneumonia 3%, it was determined by assuming the values reported on a cost-effectiveness study of PCV7 performed in Colombian children during 2010 [18]. Vaccination coverage of 90% was assumed, in line with the assumptions by other authors [18]. A herd effect of 42% was assumed based on the decline rate of IPD in infants 0 to 60 days old that were not vaccinated with PCV7 who lived in the same regions of the vaccinated children [20]. Thus, if 10% of children are not vaccinated, the protecting effect of the vaccine would also benefit 4.2% of this 10% (Table 1).

**Table 1:** **Demographic and epidemiologic parameters of the probability of developing pneumococcal disease and the efficacy of PCV13 and PCV10 and cost of care in Colombian children younger than 5 years, 2014**

**Parameters**		**Mean value**		**Data distribution**	**Reference**
**Demographic**					
Newborns in 2012		676 835		Does not vary	[19]
Life expectancy		73,78		Does not vary	[9]
Discount rate		3%		2% - 5%	[13]
**Epidemiologic**					
Pneumococcal sepsis probability		0,000184		Beta	[13]
Pneumococcal meningitis probability		0,000037		Beta	[13]
Radiographically confirmed pneumonia probability (a)		0,007441		Beta	[13,15]
Pneumococcal AOM probability (b)		0,031171		Beta	[15,17]
PID mortality (meningitis, sepsis)		37%		Beta	[18]
Pneumonia mortality		3%		Beta	[18]
Vaccination coverage		90%		Beta	Assumption
Herd effect		42%			[20]
**Parameter**	**Mean value**	**Range**		**Data distribution**	**References**
		**Inferior limit**	**Superior limit**		
**Efficacy of intervention**					
Meningitis, sepsis PCV10	65,0%	11,1%	86,2%	Beta	[22]
Radiographically confirmed pneumonia PCV10	22,4%	5,7%	36,1%	Beta	[22]
AOM due to *S. pneumoniae* PCV10 (c)	32,4%	21,6%	40,4%	Beta	[11,16]
Meningitis, sepsis PCV13	89,1%	73,7%	95,6%	Beta	[5]
Radiographically confirmed pneumonia PCV13	30,3%	10,7%	45,7%	Beta	[21]
AOM due to *S. pneumoniae* PCV13 (d)	68,1%	61,5%	74,6%	Beta	[11,23]
**Vaccine costs**					
Cost of PCV10	$ 14,12	$ 12,71	$ 15,53	Gama	[24]
Cost of PCV13	$ 15,68	$ 14,11	$ 17,25	Gama	[24]
Administration cost (per dose)	$ 1,00	$ 0,9	$ 1,10	Gama	Assumption
Cost of sepsis	$ 8 192	$292	$ 104 535	Gama	Health insurance company
Cost of meningitis	$11 595	$ 1 165	$ 54 891	Gama	Health insurance company
Cost of pneumonia	$ 1 854	$ 306	$ 40 812	Gama	Health insurance company
Cost of AOM	$ 40	$ 36	$ 44	Gama	[26,27]

a. Incidence of pneumonia in Medellin in 2009, adjusted to the proportion of pneumonia cases confirmed radiographically (Benavides et al [13]).

b. Incidence of AOM in Medellin in 2009, adjusted to the proportion of AOM cases due to pneumococcus (Sierra et al [17]).

c. Clinical efficacy of PCV11 for preventing AOM due to *S. pneumoniae*, adjusted according to the proportional frequency of serotypes circulating in Colombia between 2009 and 2012, contained in PCV10.

d. Clinical efficacy of PCV7 for preventing AOM due to *S. pneumoniae*, adjusted according to the proportional frequency of serotypes circulating in Colombia between 2009 and 2012, contained in PCV13.

### Measurement of effectiveness

Vaccines effectiveness was assessed by a systematic review The following databases were used: EBSCO Host, EMBASE, OVID, PLoS, PubMed, Science Direct, Springer, Wiley InterScience, Wolters Kluwer Health and Randomized Controlled Essays were considered. The MeSH terms used in the search, in different combinations, were: pneumococcal vaccines, streptococcus pneumonia, sepsis, pneumococcal meningitis, pneumonia, and otitis media.

The repeated references were removed, as well as those with polysaccharide vaccines; with adults with immunodeficiencies; with senior adults; with a shorter than six months follow up period and those that only evaluated immunogenicity without considering clinical results.

PCV7 was the first conjugated vaccine with published efficacy studies for IPD; PCV10 and PCV13 were approved based on immunogenicity comparisons with PCV7. One RCT determined the efficacy of PCV7 for preventing meningitis and sepsis in children under 5 years [5]. The PCV13 efficacy for radiologically-confirmed pneumonia was taken from a study made with PCV7 in children under than 5 years [21].

The PCV10 efficacy for radiologically-confirmed pneumonia and IPD was taken from the COMPAS study, a multicenter study in which Colombia participated [22].

The effectiveness of vaccines against AOM, were adjusted according to the prevalence of the pneumococcus serotypes in Colombia. Based on their clinical effectiveness, this was adjusted according to the circulating serotypes in the country and that are contained in each vaccine. We did that adjustment because a vaccine could have a high effectiveness but against serotypes with a low prevalence in our country.

In the case of PCV13, we took PCV7 efficacy to prevent pneumococcal AOM (83% (IC 95%: 75% - 91%)) [23] caused by serotypes contained in PCV7. To determine the efficacy of PCV10 to prevent pneumococcal AOM, we assumed it reported on a RCT that evaluated an 11-valent precursor to PCV10 (52.6% (IC 95%: 35.0% - 65.5%)) [16]. Based on the reports by SIREVAII between 2009 and 2013, PCV10 covers 61.6% and PCV13 covers 82.0% of pneumococcus serotypes causing invasive disease in Colombia in children under than 5 years [11]. It means, PCV13 efficacy was adjusted by 82.0% and PCV10 efficacy was adjusted by 61.6% (Table 1).

### Economical parameters

The direct costs include the costs of the PCV10 (Synflorix®) and PCV13 (Prevenar 13®) vaccines and the application of three doses. The values used are the ones recommended by the Pan American Health Organization for 2014 [24]. Medical costs associated with the treatment of disease were assumed to be a lower value to pay for each case prevented (Table 1).

The database of a national health insurance company which has more than 2 million members (which ensures the representativeness of the population), was used to determine the costs for treating sepsis, meningitis and pneumonia in children younger than 5 years in Colombia. The costs were determined based on the value paid by the health insurance companies to the hospitals and it comprises all of the services offered during the hospitalization including hospital visits, diagnostic aids, antibiotic treatments and other necessary services used for the recovery of the patient until discharge. The rates for all these services are based on a government reimbursement manual for national application. In 2013, this health insurance company reported a total of 72 children younger than 5 years with sepsis, 36 with meningitis and 541 with pneumonia. These values were adjusted with the Consumer’s Price Index reported by the DANE for 2013: 1.94% [25]. An exchange rate of COP 1 959 was assumed for the calculation in dollars, since it was the average exchange rate for the first six months of 2014.

To calculate the cost of care for an episode of AOM, two clinical guidelines [26,27] that propose the same antibiotic treatment (amoxicillin 90 mg/kg/day) were considered. The cost of a 90 ml acetaminophen bottle was added to this treatment regimen as well as the cost of two outpatient visits, based on the values of the national tariff manual updated to 2014 [28] (Table 2).

**Table 2:** **LYG, Total costs and ICER for PCV13, PCV10 and no vaccination in Colombian children younger than 5 years, 2014**

	**No vaccine**	**PCV10**	**PCV13**
Total discounted LYG	0	18 708	25 396
Discounted medical costs avoided	0	$ - 13 703 271	$ - 19 479 395
Vaccine costs*	0	$ 28 920 564	$ 31 904 432
Total costs	0	$ 15 217 293	$ 12 425 037
**C/E Prevented cases** (per additional LYG)		$ 813.41	$ 489.26
**Incremental analysis**			
ICER No Vaccine vs. PCV10	$ 813.41		
ICER PCV10 vs. PCV13	$ - 417.53		

*Included administration costs.

LYG: Life Years Gained.

C/E: Cost-effectiveness ratio.

It was considered that an alternative is cost-effective if the cost per additional unit of effectiveness (LYG) is less than 3 Colombian GDP per capita (US$ 24,075 for 2013), and highly cost-effective if less than 1 Colombian GDP per capita (US$ 8 025 for 2013) [29].

### Incremental cost-effectiveness analysis

When a technology is more cost-effective than other but at the same time is costlier, it is necessary an economic decision criteria. The question in this case would be how much additional costs have to be paid for the additional effectiveness. This is known as the Incremental Cost-Effectiveness Ratio (ICER). In other words, what was the incremental cost for PCV13 compared to the PCV10 and not vaccination related to the corresponding incremental effectiveness?

### Sensitivity analysis

In order to assess the robustness of the model, as well as the sensitivity of the ICER to the parameters of the main variables, univariate and probabilistic sensitivity analysis were done. For the first case, the extreme values of the ranges were used to test the ICER results. For the probabilistic sensitivity analysis, a one thousand iterations Montecarlo simulation was performed. Beta distribution was used for clinical parameters and gamma distribution for the avoided medical costs and vaccine costs. The beta and gamma parameters were calculated with the respectively means and standard deviations of each variable. Acceptability curves and cost-effectiveness plane was included for measurement uncertainty reflected by the model.

## Results

### Effectiveness

After 5 years follow up projection, compared to no vaccination, PCV13 would prevent 66 005 pneumococcal AOM cases and 437 deaths due to pneumococcal infections and PCV10 would prevent 31 401 pneumococcal AOM cases and 321 deaths due to pneumococcal infections. In the same way, PCV13 would generate 25 396 LYG and PCV10 would generate 18 708 LYG (Table 3).

**Table 3:** **Prevented cases of sepsis, meningitis, pneumococcal AOM and radiographically confirmed pneumonia; as well as prevented deaths per year and life years gained, when applying PCV13 and PCV10 in Colombian children younger than 5 years, 2014**

**Parameter**	**PCV13**	**PCV10**
**Prevented cases**		
Pneumococcal sepsis	510	372
Pneumococcal meningitis	103	75
RX confirmed pneumonia	7 011	5 183
Pneumococcal AOM	66 005	31 401
**Prevented deaths**		
Pneumococcal sepsis	189	138
Pneumococcal meningitis	38	28
RX confirmed pneumonia	210	155
All causes	437	321
**Life years gained**		
Pneumococcal sepsis	5 565	4 059
Pneumococcal meningitis	1 119	816
RX confirmed pneumonia	18 712	13 832
All causes	25 396	18 708

### Costs

Although the cost to vaccinate 90% of children was higher for PCV13 compared to PCV 10 (US$ 31 904 432 versus US$ 28 920 564), this price is compensated by the greater savings obtained by the avoided cases with PCV13 compared to PCV10. Medical costs avoided were US$ 19 479 395 for PCV13 and US$ 13 703 271 for PCV10.

### Cost-effectiveness of the pneumococcal conjugate vaccines

PCV13 showed a better cost-effectiveness ratio for the cases of meningitis, sepsis, radiologically-confirmed pneumonia and AOM prevented. Cost-effectiveness ratio to prevent pneumococcal AOM cases was US$ 446.15 with PCV13 and US$ 883.79 with PCV10. Compared to no vaccination, the ICER of PCV13 US$ 489.26 per LYG, while the ICER of PCV10 was US$ 813.44 per LYG. With a greater effectiveness and lower costs, PCV13 was dominant to PCV10 (Table 2).

### Univariate sensitivity analysis

**Figure 2:**
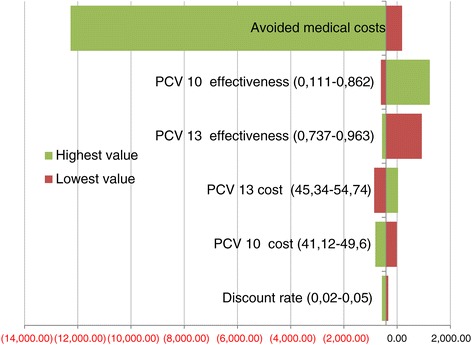
**Tornado diagram of PCV13 vs. PCV10 to prevent pneumococcal diseases in a cohort of Colombian children, 2014.**

For the univariate sensitivity analysis, the high and low ranges of the discount rate, vaccines’ costs, treatments costs and effectiveness of the vaccines were tested in the univariate analysis. It allows to observe how sensible was the ICERs to this changes. PCV13 was dominant over PCV10 in ten of 12 variations (Table 4 and Figure 2). Therefore, the ICER result in the univariate sensitivity analysis shows a very robust model.

**Table 4:** **Univariate sensitivity analysis of ICER results of PCV13 vs PCV10 vs no vaccination in Colombian children younger than 5 years, 2014**

	**Base case**			**Lowest value**			**Highest value**		
	**Base case value**	**ICER**		**Value to prove**	**ICER**		**Value to prove**	**ICER**	
		**NV vs PCV10**	**PCV10 vs PCV13**		**NV vs PCV10**	**PCV10 vs PCV13**		**NV vs PCV10**	**PCV 10 vs PCV13**
Discount rate	3%	813,41	- 417,53	2%	612,22	- 338,43	5%	1.288,37	- 572,40
**Vaccine costs (US$)**									
PCV10	45,36	813,41	-417,53	41,12	669,04	- 13,68	49,60	957,77	- 821,39
PCV13	50,04	813,41	- 417,53	45,34	813,41	- 866,01	54,74	813,41	- 30,94
**Medical costs (US$)**									
Avoided cases	Mean values	813,41	- 417,53	Mean values	1.401,61	- 185,47	Mean values	11.082,66	- 12.268,58
**PCV10 effectiveness**									
Sepsis	0,6500	813,41	-417,53	0,1110	5.804,26	- 609,93	0,8620	288,94	- 1.223,03*
Meningitis	0,6500			0,1110			0,8620		
Pneumonia	0,2240			0,0570			0,3610		
OMA	0,3240			0,2160			0,4040		
**PCV 13 effectiveness**									
Sepsis	0,8910	813,41	- 417,53	0,7370	813,41	- 923,48*	0,9630	813,41	- 568,52
Meningitis	0,8910			0,7370			0,9630		
Pneumonia	0,3030			0,1070			0,4468		
OMA	0,6810			0,6150			0,7944		

PCV13: 13-valent pneumococcal conjugate vaccine.

PCV10: 10-valent pneumococcal conjugate vaccine.

NV: no vaccination.

*In these two cases, PCV10 is dominant.

### Probabilistic sensitivity analysis

**Figure 3:**
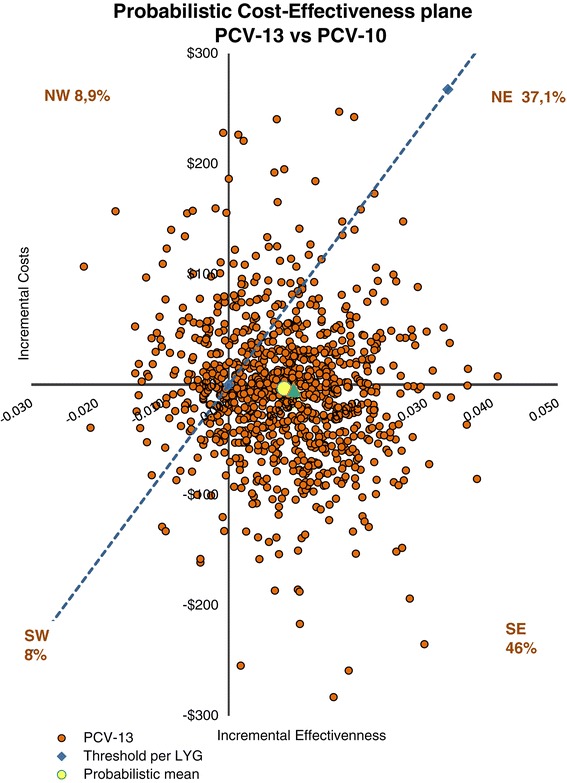
**Probabilistic cost-effectiveness plane in which the LYG are plotted versus the costs for PCV13 and PCV10 in a cohort of Colombian newborns, 2014.**

The probabilistic sensitivity analysis showed the robustness of results of the model. Most of the simulations of PCV10 and PCV13 overlapped each other, and were located in the NE and SE quadrants relative to not vaccinating, supporting that both options were more effective than not vaccinating. PCV13 offered more LYG than PCV10 and a lesser cost. In 81% of the 1 000 simulations made by the model, PCV13 is cost-effective compared to PCV10 (Figure 3).

### Acceptability curves

**Figure 4:**
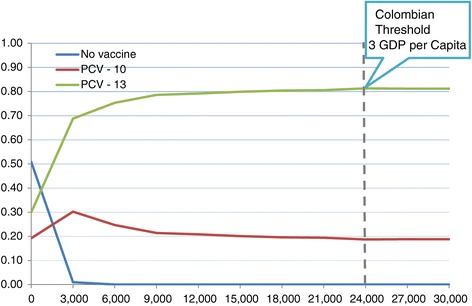
**Cost-Effectiveness Acceptability curves of PCV13 vs. PCV10 vs. no vaccine to prevent pneumococcal diseases in a cohort of Colombian children, 2014.**

The acceptability curves allow to know what is the likelihood for a strategy to be chosen, compared with the another one. For a willingness to pay of 1 GDP per Capita for Colombia, PCV13 has a probability of 78% to be chosen, meaning this is strategy is highly cost-effective. In the same way, for a willingness to pay of 3 GDP, PCV13 has a probability of 81% to be chosen and PCV10 has a probability of 19% (Figure 4).

## Discussion

This cost-effectiveness analysis has identified the clinical and economical results of PCV13 and PCV10. From the Colombian health system perspective both strategies are superior to no vaccination in terms of prevented cases, ICER, LYG and prevented deaths.

PCV13 presented more benefits in regards to prevented cases and deaths due to IPD, radiologically-confirmed pneumonia and AOM. PCV13 is the dominant option over PCV10 since it avoids more deaths, more LYG are obtained and fewer resources are consumed due to greater reduction in disease.

There is no doubt that vaccination with pneumococcal vaccines from 2000 has been a positive impact on public health to prevent pneumococcal diseases, particularly mortality related with *S. pneumoniae.* Our main contribution to this discussion is to generate valid information that allows decision makers in Colombia, to identify which of the strategies available is more cost-effective to carry out an immunization program with pneumococcal vaccines in our country.

On 2007 PCV7 was included in the Colombian Plan of Immunization; initially for high risk population and later for low weight newborns and the ten departments with the highest mortality rates [30]. Since 2010, was implemented the universal vaccination with PCV13 based on the additional coverage of six serotypes increasing the coverage up to approximately 60% of the national territory. In September 2011 the Colombian Plan of Immunization replaced the PCV13 with PCV10. This change was after the publication of a cost-effectiveness study that evaluated PCV7, PCV10 and PCV13 [19]. There was a model that assumed that PCV10 would have an excess impact on preventing AOM due to all causes, even though PCV13 would prevent more cases of pneumonia, IPD and deaths [18].

The main differences of this model with the latest cost-effectiveness study published on the subject in the country [18] are (1) the assumption that PCV10 is effective against non-typeable *H. influenza,* (2) the AOM prevalence parameter which was not adjusted specifically for pneumococcus, (3) the medical costs source is not local and (4) the time periods analyzed in both studies were different (2007-2009 vs. 2009-2013).

The higher number of IPD and pneumonia cases avoided with PCV13 shown in both studies was based mainly on the better coverage of the circulating serotypes in Colombia by PCV13; serotype coverage is 33% higher than the coverage by PCV10: 82.0% vs. 61.6% [11].

Although the cost of every dose of PCV13 is US$ 1.56 higher than PCV10, the largest IPD and AOM cases avoided with PCV13 allows the latter to be a dominant strategy, because the savings generated by direct costs of treatment of cases avoided are greater than the costs difference in an immunization program between both of them strategies. Even though the analysis perspective was from a Colombian health system, it could be inferred that from a societal perspective the results would be similar, because issues related with both of patients and caregivers, such as travel times to medical appointments or time to care sick children, will be greater in the strategy that avoided fewer IPD and AOM cases.

The results of this research coincide with another ten cost-effectiveness analysis on PCV13 performed in different countries [31-40]. All these conclude that PCV13 prevents more cases and more deaths due to pneumococcal diseases than PCV10 and thus is a better alternative in terms of costs compared to PCV10. Strutton et al [31] conclude that a pediatric national immunization program with PCV13 would eliminate 31.7% of IPD in Germany, 46.4% in Greece, and 33.8% in the Netherlands, and it was a cost saving strategy, compared with PCV7 and PCV10. Earnshaw et al point out that an immunization program with PCV13 is a cost-saving strategy in Canada because it provides substantial public health and economic benefits versus to PCV10 [33].

Furthermore, Newall et al assert that the high proportion of current IPD caused by serotype 19A (included in PCV13 but not in PCV10) may be an overriding factor in the design of vaccination policies in Australia [32]. In the same way, isolates in Colombia of serotype 19A have increased seven times, and serotype 3 have increased four times, between 2009 and 2013 compared with the period between 1994 and 2008 [11].

In the same way, all these health economic evaluations [31-40] used analytical decision tree models as the best option to explain the relationship between the natural history of pneumococcal diseases and PCV, because these are acute diseases and their probability of recurring events is low. In fact, the effectiveness of both vaccines used in this model is calculated only against the serotypes included in each vaccine and we did not considered cross immunogenicity. Although there is the likelihood to suffer disabilities in patients with meningitis, i.e. deafness, our main outcomes were mortality and life years gained, since this is a cost-effectiveness analysis, not a cost-utility analysis.

In Latin America there are two important studies with similar results, one in Mexico and another one in Uruguay. The first one concludes that immunization with PCV7, PCV10 or PCV13 would be cost-saving interventions, however, health outcomes and savings of the strategy with PCV13 are greater than those estimated for PCV7 and PCV10 [37]. The later concluded that there was a significant decline on incidence of hospitalizations for consolidated pneumonia in children younger than 2 years of age, related with a vaccination schedule of 2 + 1 with PCV13 [40].

Five research studies have concluded that PCV13 avoids more deaths due to pneumococcal diseases than PCV10, but no more cases of overall disease [18,37,41-43]. The results of these differ from the above studies because they assume that PCV10 is effective in preventing AOM due to *S. pneumoniae* and also to non-typeable *H. Influenzae.* Such cost-effectiveness analysis based this assumption on the study made on PCV11 by Prymula [16]. However, this study did not use PCV10, used a 3 + 1 immunization schedule, and represented only a subset of the most severe cases of AOM with a higher proportion of bacterial cases.

A recent study showed PCV10 to have considerably less impact on all cause AOM in a controlled environment particularly when used in a 2 + 1 schedule [44]. By contrast, PCV13 is similarly effective in 3-dose and 4-dose schedules versus AOM [45]. In addition, a recent randomized trial of NP carriage of NTHi comparing PCV7 to PCV10 found no effect of PCV10 in reducing NTHi carriage. Therefore, it is unlikely that any proposed non-pneumococcal benefits will be realized with PCV10 [46]. Furthermore, the regulatory approval issued by the INVIMA and the European Medicines Agency points out that PCV10 is indicated for preventing diseases produced by the serotypes included in the pneumococcal vaccine [47].

Results from the Clinical Otitis Media and Pneumonia Study (COMPAS) with PCV10 could not provide definitive evidence on how the marketed formulation can impact all-cause otitis media in a Latin American setting, because they had a lower than expected number of AOM cases [23].

Based on pneumococcal effectiveness and serotype distribution locally, PCV13 is cost-saving versus PCV10 in regards to preventing pneumococcal disease due to all causes, preventing mortality and obtaining LYG, which leads to lower costs of care.

The main strength in this study is the local information which helps to determine the epidemiological parameters for Colombia in regards to the diseases of interest [10,11,13,15,17,19]. Likewise, the costs of the medical treatments are based on a national third-party payer, based on what really got paid to hospitals for the care of patients. There was no underreporting of the costs information (since it is the universe of the data base of what was paid by the insurer). The adjustment of the effectiveness of both vaccines based on the pneumococcus serotypes circulating in Colombia between 2009 and 2013 permits an updated analysis. Finally, this study includes information on the herd effect of PCV7 observed in children [20], assuming that such effect is similar for both vaccines.

The main weakness of this study is we do not have information from a head to head clinical trial between PCV13 and PCV10. Similarly, results of the COMPAS [22] study are difficult to compare because the average of follow up was 23 months, compared to four and a half years of the *Northern California Kaiser Permanente Vaccine Study* [5]. It could affect the results of the first one trial because the follow up was 31 months lesser than *Kaiser Study,* but is the best information source available about PCV10 effectiveness. In the same direction, lack of inclusion of a herd effect for children > 5 years of age and adults underestimates the full potential impact of the vaccines, in particular the additional benefit of PCV13, because serotypes 3, 6A and 19A cause pneumococcal disease in older adults.

Likewise, because the data on the prevalence of disease was taken from official records, some underreporting could have taken place, since the information depends on whether or not the physician used the correct diagnosis code. In the same way, the burden of diseases by pneumonia could have been underestimated, because we just considered cases of radiologically-confirmed pneumonia.

This study did not evaluate aftermath generated by any of the diseases, this is why the quality-adjusted life years was not determined. It must be emphasized that in terms of prevention of death due to all causes PCV13 was dominant over PCV10. Further, PCV13 prevented more cases of disease, including meningitis, and therefore would be expected to provide greater reduction in LYG and thus remain dominant in analysis.

## Conclusion

PCV13 and PCV10 are better cost-effectiveness alternatives compared to no vaccination. PCV13 is dominant over PCV10 since it prevents more deaths, generates more LYG and the expected costs are lower (ICER PCV10 vs. PCV13: US$ -417.53).
